# Dynamics of single mothers’ semantic strategies in Anglophone non-fiction

**DOI:** 10.12688/openreseurope.15729.2

**Published:** 2023-08-08

**Authors:** Dmitrii Sergeev

**Affiliations:** 1 Faculty of Education, University of Cambridge, Cambridge, CB2 8PQ, UK

**Keywords:** single motherhood, non-fiction, self-help, sematic strategies, gender identification, critical discourse analysis, meaning-making, cultural semantics

## Abstract

The article discusses how the increasing number of self-help books for single mothers is meeting the demand for positive narratives about their experiences, which are often stereotyped in the media. The books offer a powerful tool for self-reflection, and content analysis reveals semantic strategies that authors use to construct, negotiate, and reframe single motherhood. The authors aim to challenge societal expectations and redefine what constitutes a family. There are still conflicting messages that perpetuate negative stereotypes and stigmas about single mothers, propelling identity negotiation. The seven principal semantic strategies applied in the texts include reframing, renaming, normalization, direct or indirect confrontations, distinctions, self-labelling, and vernacular. By using semantic strategies, authors aim to empower single mothers, challenge negative stereotypes, and create a sense of community. While there are limitations to the self-help genre, it remains a powerful tool for self-reflection, self-empowerment, and the construction of identity.

## Plain language summary

This article talks about how self-help books for single mothers are becoming more popular because they provide positive and empowering narratives about their experiences. Single mothers are often stereotyped in the media, and these books help to challenge those stereotypes and redefine what a family looks like. Through content analysis, the authors of the article identified seven semantic strategies that authors use to construct, negotiate, and reframe single motherhood, including reframing, renaming, normalization, direct or indirect confrontations, distinctions, self-labelling, and vernacular. By using these strategies, authors aim to empower single mothers, challenge negative stereotypes, and create a sense of community. While there are limitations to the self-help genre, it remains a powerful tool for self-reflection, self-empowerment, and the construction of identity.

## Introduction

The increasing number of self-help books for single mothers reflects a desire for positive narratives about this highly stereotyped experience ( Bock, 2000). Since the emergence of self-help literature “as an icon of popular culture” ( Cherry, 2008, p. 337) in the last century, the genre has attracted the attention of researchers ( Starker, 1990). Much attention has been drawn to advice literature in the United States, the country with most advanced self-help culture ( Archibald, 2007). Wendy Simonds investigated the appeal of self-help books and what they reveal about gender relations in contemporary American culture. She analyzes bestsellers since 1963, including books on relationships, self-esteem, and spirituality, and argues that the success of the genre reflects readers’ search for meaning. Simonds examines how self-help books affect the social construction, cultural consumption, and transmission of ideas about gender and the self ( Simonds, 1992).

Scholars have conducted surveys in attempt to trace the history of advice books for women ( Urban, 2006) and to discern the portrayals of different types of motherhood, such as stepmothers, within this genre ( Renegar & Cole, 2019). Renegar and Cole have identified common trends in the representation of mothers in self-help books that tend to comply with heteronormative requirements because these books are often “written by white, cisgender, heterosexual, and affluent women who have transitioned from successful single women to stepmothers” (2019, p. 512).

Although self-help books have been continually criticized as reinforcing repressive societal values about women ( Cherry, 2008; Ebben, 1995; Grodin, 1991; Simonds, 1996), we cannot ignore their potential for transformation of self-identity and social relationships ( Nehring *et al.*, 2016, p. 19). Taking into consideration that single mothers were ostracized across mass media ( Salter, 2018), self-help might be regarded as a powerful and sought-after medium for vocalizing their own experiences, negotiating their identity, and constructing a more personalized image of themselves. Printed texts remain a powerful tool for constructing self-images, resisting social pressures, or, conversely, complying with their force. Even in the era of ever-expanding and ubiquitous internet and IT, self-help books serve the purpose of self-reflection about personal experiences in a detached style, allowing individuals to share their personal and intimate life experiences through an objectifying literary genre. Non-fiction provides authors with a cultural instrument to step out of their insider position and become a narrating, judging outsider. This allows them to reflect on their own community in an attempt to strengthen its social position or, contrarily, to detect any differences and distance themselves from what may appear to be a seemingly homogeneous group.

It is worth noting that in this paper, the notion of a single mother derives from the analysed texts and aligns with today’s broader understanding of single motherhood ( Letablier & Wall, 2018). Therefore, a single mother is defined as a woman who raises a child or children without a partner in the household. This definition includes self-evident cases of chosen single motherhood, widowhood, divorced motherhood, or motherhood resulting from pregnancy out of wedlock. Additionally, a mother having a partner who lives separately and is regularly or irregularly involved in fostering a child is also considered as single.

## Methods

### Source selection

I compiled the corpus by applying the filters “non-fiction” and “single mothers” on Amazon.com. I compiled a list of all books suggested by Amazon and then refined them through a selection process using the following three essential criteria: (1) self-help books as a genre (2) written by English-speaking single mothers and (3) published between 1990 and 2020. I therefore excluded a psychological non-fiction written by a male author. The final non-specified list included 30 books. They represent subgenres of self-help: 14 traditional self-help books, four that function similarly to self-help styled devotionals with numbered tips for survival, 11 that are a combination of self-help and memoir, and one that is a combination of self-help and research. All of the authors assert that they have experienced single motherhood either as a prolonged state or for some period in their lives.

### Data collection

During the initial stage of data collection, an Excel chart was created to track the formal features and semantic patterns of the books being considered. This chart included the year of the first publication, the country of publication, the authors’ ethnical self-identity, the category of single motherhood, the type of self-help narratives, self-perception and self-portrait, social institutions, social surroundings, social class of the authors, discussed issues, and mothering strategies. These categories were chosen to provide a comprehensive understanding of the books and their authors.

Moving forward, the next stage involved classifying the nouns and adjectives used by the authors to describe themselves and to discuss social strategies for addressing every day and global issues. The authors’ language choices provided me with valuable insight into their perspectives and experiences as single mothers.

Overall, the data collection process was carefully designed to capture important information about the books and their authors. By tracking formal features and semantic patterns, and by analyzing the language used by the authors, I was able to develop a nuanced understanding of the narratives and their strategies.

The complete list of self-help books includes:

Adams J: Complete Guide for New Single Moms. Ocala, FL: Atlantic Publishing Group,2011.

Bailey R: Motherhood My Way: Becoming a Single Mother by Choice. Scotts Valley, CA: CreateSpace,2018. 

Brockes E: An Excellent Choice: Panic and Joy on My Solo Path to Motherhood. London: Faber and Faber,2018.

Brown AE: The Single Mother’s Diary: 10 Women Who Have Defied the Odds of One Parent Homes. Highlands Ranch, CO: Authors Place Press,2019. 

Conteh-Mosere S: Single Mother Breaking: The Stereotype. Milton Keynes: ESCIEM Publishing Group,2017.

Corder H: The Successful Single Mom: Get Your Life Back and Your Game On! Nashville, TN: Honoree Enterprises Publishing,2009.

Edgar NL: All Things Beautiful: 31 Devotions for Single Moms. Savage, MN: BroadStreet,2018.

Farr N: I am the Parent Who Stayed: Joyfully parenting alone. London: Practical Inspiration Publishing,2017.

Floch C: The Single Mom’s Devotional: A Book of 52 Practical and Encouraging Devotions. Grand Rapids, MI: Revell, [2010]2014.

Golds A: How To Be Your Own Boss As A Single Mum. Scotts Valley, CA: CreateSpace, 2014.

Hanscome J: Suddenly Single Mom: 52 Messages of Hope, Grace, and Promise. Franklin, TN: Worthy Inspired, 2016.

Hertz R: Single by Chance, Mothers by Choice: How Women are Choosing Parenthood without Marriage and Creating the New American Family. New York, NY: Oxford University Press, 2006.

Hudson H: The Badass Single Mom’s Survival Guide: 21 Life Hacking Tips. Scotts Valley, CA: CreateSpace,2017. 

Isenhart D: Surviving Single Parenting. Bloomington, IN: iUniverse, 2000. 

Johnson E: The Kickass Single Mom: Be Financially Independent, Discover Your Sexiest You and, Raise Fabulous, Happy Children. New York, NY: Tarcherperigee, 2017.

Kowalski S: Motherhood Reimagined: When Becoming a Mother Doesn’t Go As Planned. Berkeley, CA: She Writes Press. 2017.

Mattes J: Single Mothers by Choice: A Guidebook for Single Women Who Are Considering or Have Chosen Motherhood. New York, NY: Three Rivers Press, 1997.

Morrissette M: Choosing Single Motherhood: The Thinking Woman’s Guide. New York, NY: Houghton Mifflin Company, 2008.

Nickell A: Confessions of a Single Mum: What It’s Like When You’re Expecting The Unexpected. London: Headline, 2018. 

Pagalday S: Who Will Hold Me?: A Single Mother’s Memoir of Self-Love, Empowerment and Freedom. Milton Keynes: Lightning Source, 2019.

Panettieri G, Hall PS: The Single Mother’s Guide to Raising Remarkable Boys. Avon, MA: Adams Media, 2008.

Reed S: Fix It Jesus! For Single Moms Only: The Straightforward No-Nonsense Guide To Passion, Purpose, and Prayer. Scotts Valley, CA: CreateSpace, 2018.

Roberts G: Going Solo: My Choice to Become a Single Mother Using a Donor. London: Piatkus, 2019.

Seney-Williams K: Surviving and Thriving on the Single-Parent Journey: A Step-by-Step Approach. Oxford: Lion Books, 2019.

Senters ML: The Unseen Companion: God With the Single Mother. Chicago, IL: Moody Publishers, 2017. 

Sloan L: Knock Yourself Up: No Man? No Problem: A Tell-All Guide to Becoming a Single Mom. New York, NY: Avery Trade, 2007. 

Smith V: The Single Mum’s Survival Guide: How to Pick Up the Pieces and Build a Happy New Life. New York, NY: Morgan James Publishing, 2014. 

Soiseth A: Choosing You: Deciding to Have a Baby on My Own. Berkeley, CA: Seal Press, 2008. 

Welch D: Love Factually for Single Parents and Those Dating Them. Eugene, OR: Love Science Media, 2019. 

Whitehurst T: God Loves Single Moms: Practical Help for Finding Confidence, Strength, and Hope. Grand Rapids, MI: Revell, 2010.

Some texts cover topics related to single motherhood in much greater detail. The corpus is arranged chronologically, allowing for a trace of the dynamics of discussions within the single motherhood community and the subsequent development of single mothers’ self-perception. Certainly, there is a cluster of texts specifically devoted to addressing issues of single mothers by choice ( Hertz, 2006; Mattes, 1997; Morrissette, 2008; Roberts, 2019) where the fertility issue, problems of inseminations and donor choice are brought forward. But even this subset of books becomes fragmented in discussing similar challenges within different demographics, such as lesbian White American single mothers by choice ( Sloan, 2007), Canadian nationals residing in New York ( Soiseth, 2008), African Americans ( Bailey, 2018), and lesbian White British residing in New York ( Brockes, 2018). Sarah Kowalski analyzes her path to single motherhood by choice as a Buddhist and advocate of alternative medicine.

The authors are from or have connections to the United States, the United Kingdom, Canada, and Jamaica, either as their place of birth or as a place to which they have migrated. The majority of the authors (21 out of 30) are from the United States, while only seven are from the UK, one from Jamaica and one from Canada. The categories of single mothers include those who are divorced (16), single by choice (10), and unwed (4). The data covers a range of years of publication, with the majority of the books published in the 2010s: 2017 – 6 books, 2018 – 5 books, 2019 – 5 books. Unsurprisingly, the majority of authors are white (26), the rest of authors self-identify as African American (2), Black British (1), and Jamaican (1). However, the authors’ self-identifications are more diverse, including other fractions such as lesbian (2) or visually impaired (1). Even the gamut of ethnical and national identifications represents an intricated pictures of a Canadian national residing in the United States (1), an American national residing in the United Kingdom (2), a British national residing in the United States (1), a Jamaican national residing in the United Kingdom (1).

### Data analysis

I applied the analytical approach, consisting of content analysis of the books’ coverage and identification of the authors’ discursive and rhetorical strategies ( Carvalho, 2008). These components of narrative structure I put under the umbrella term “semantic strategy”.

My broad analysis of self-help books focuses on how they contribute to the formation of identity within the single motherhood community, and how they can either reinforce or challenge stereotypes. Specifically, I examine the semantic strategies that authors / single mothers use to define their subjectivity, and the impact of these strategies on shaping the identity of single mothers. Additionally, I investigate how these self-help books provide a platform for single mothers to share their personal experiences and construct their own narratives.

The implementation of content analysis in this research means to track and assess value-laden nouns, adjective and expression which fall into one of the following semantic fields: social norms; stereotypes; self-description; pregnancy; family and kinship; mothering. Interfiled examination allowed me to specify linguistic, rhetorical, discursive and narrative techniques the authors used to describe their overall single motherhood experience.

## Results

The reviewed books follow the typical self-help format of presenting a problem and providing a solution. They cover a wide range of issues related to single motherhood that can be roughly divided into broad categories: everyday life issues, professional and personal development, legal aspects of single motherhood, mothering strategies, and single mothers’ well-being. Classification itself and thorough discussion of these issues are beyond the scope of this paper and would require separate research. The list of topics, if being put together, is extensive and includes finance planning and budgeting, allocation of time and priority of simultaneous activities, conception methods, miscarriage, and dealing with hospital personnel and visitors during the postpartum period. Additionally, the books discuss challenges beyond a woman’s ability to impact directly, such as stereotypes, discrimination, or societal stigmas. Some texts also address topics like bolstering low self-esteem and overcoming guilt toward children. One recurring theme across all the literature in the corpus is the importance of developing a support network of people who can provide assistance in dealing with both minor and significant issues of single motherhood.

Closer to 2020, the diversity of self-help books became even more obvious ( Conteh-Mosere, 2017; Corder, 2009; Edgar, 2018; Hanscome, 2016). Jeanette Hanscome discusses challenges she addressed as a White American divorcee with visual impairment (2016). Ava Eagle Brown shares Jamaican-born single mothers’ stories of professional and personal success (2019). Gina Panettieri puts particular stress on issues related to raising boys without a permanent male partner in the household ( Panettieri & Hall, 2008). Ali Golds reiterates the steps necessary to set up a business (2014). Nina Farr shares her personal story of an abusive relationship and how single motherhood was an escape and respite from it (2017). It is interesting to note that advice books authored by divorced single mothers exhibit a sense of solidarity within the entire community, aiming to assist all types of single mothers ( Adams, 2011; Golds, 2014; Johnson, 2017; Panettieri & Hall, 2008; Welch, 2019).

The disproportionate representation of white, Christian, heterosexual authors reflects the targeted audience of the analysed self-help books, which is highly likely to be consumed by white, privileged women. Additionally, a significant proportion of divorced single mothers among the writers might also confirm the dominance of biological privilege narratives.

## Discussion

***Identity negotiation.*** I argue that the non-fiction narratives of single mothers replicate persistent contradictions at their core. On the one hand, they seek to challenge existing societal norms by constructing their individual and community identities in opposition to society. On the other hand, single mothers’ non-fiction narratives also reaffirm their conventionality ( Brockes, 2018, p. 11; Hertz, 2006; Mattes, 1997, p. 124). This necessity to find a middle ground might create a formally illogical statement: "In fact, many have made the decision to bear a child out of wedlock because they respect marriage too much to enter into it lightly for reasons of social and procreational expediency" ( Sloan, 2007, p. xiii).

In a narrative battlefield to face opposition and criticism from society, self-help authors resist existing social norms and construct their own definitions of family and parenthood. They are often forced to operate outside the realm of convention and chart new territory, paving the way for future generations of parents. The strategies, too, allows single mothers to assert their individual and community identity. The nonfiction writers challenge the existing social norm that single mothers must adhere to a certain standard. Emma Johnson acknowledges the effort single mothers put into pushing the boundaries of what is deemed acceptable in society ( Johnson, 2017, p. 204), and credits them with charting new territory in parenting ( Johnson, 2017, p. 240). This sentiment is echoed in Sarah Kowalski’s idea, which emphasizes the freedom and agency that single mothers have in defining their own families outside of conventional norms: “I could construct whatever I wanted. There were no limits” ( Kowalski, 2017, p. 53). Additionally, Amy Nickell underlines the fact that being a parent is not defined by marital status, and that unconventional family structures can be just as fulfilling and complete as traditional ones. These statements challenge the societal assumption that a two-parent household is necessary for a child’s well-being. Amy Nickell dismisses the notion that an unplanned baby can ruin women’s life, and highlights that single mothers’ ability as a parent is not defined by marital status ( Nickell, 2018, p. x, 89, 100). Mikki Morrissette stresses the importance of recognizing that a home without a father is not the same as a home without values ( Morrissette, 2008, p. 136). It is essential to acknowledge that a stable and loving home environment is crucial for a child’s development, regardless of the family structure. Overall, they all underline the need to challenge societal expectations and redefine what constitutes a family.

Single mothers navigate the fine line between accepting and rejecting societal stereotypes in their narratives, often attempting to reject social pressure while also sometimes inadvertently reproducing negative stereotypes about themselves. A critical aspect of these discussions is the outsized influence of the regime of intensive mothering – a dominant cultural narrative that prescribes a highly demanding and hands-on approach to any types of motherhood. This regime not only shapes societal expectations but also places immense pressure on single mothers to conform to specific standards of nurturing, involvement, and sacrifice. The discourse surrounding intensive mothering plays a significant disciplining role, compelling single mothers to align with its ideals and punishing those who deviate from the prescribed path. An insightful examination of the disciplining function of the regime of intensive mothering sheds light on how it operates as a potent mechanism, shaping their choices and behaviours. Therefore, the self-help authors are bombarded by contradictory messages which they transmit in their own narratives. Women are pressured to be superwomen ( Pagalday, 2019, p. 60, 73), to do everything perfectly, and to take on both the mother and father roles. However, at the same time, they are told that it’s okay to have doubts, that solo parenting is not for perfectionists ( Roberts, 2019, p. 219), and that they cannot be superwomen ( Isenhart, 2000 p. 77; Panettieri & Hall, 2008, p. 97; Senters, 2017 p. 57; Whitehurst, 2010, p. 17). This contradiction can create confusion and conflict in the minds of single mothers as they try to live up to society’s expectations while dealing with the challenges of solo parenting on an everyday basis.

The conflicting messages therefore create a ground for single mothers’ identity negotiation. They may struggle to define themselves as mothers and individuals, as they navigate the societal expectations of what it means to be a mother and a single parent. One of the most pronounced confusions reappears in the contradiction of seemingly simultaneous possibility and impossibility of being a perfect mother ( Adams, 2011, p. 71, 185; Farr, 2017, p. 134; Hudson, 2017, p. 43; Panettieri & Hall, 2008, p. 88, 134). Kat Seney-Williams presents this confusion in a more individualised way as surviving – thriving (2019, p. 22).

Moreover, these contradictions can also support negative stereotypes and stigmas attached to single mothers. The idea of a “superwoman” can perpetuate the myth that single mothers do not need help or support, and they are self-sufficient. At the same time, the idea that they cannot be superwomen can reinforce the stereotype that single mothers are weak or incompetent.

The identity negotiation can manifest narratively through a multitude of questions that they are expected to answer ( Seney-Williams, 2019, p. 177) in various ways. This negotiation can trigger a rediscovery of positive self, or at least a reflective search for renewed and more comfortable self-identity. As Alexandra Soiseth (2008, p. 260) writes, “I knew that having a baby would make me see the world in a new way; I just didn’t realize how much it would make me see myself and my relationship with my parents in a new way.” For some, being their authentic selves is key, as Holly M. Hudson (2017, p. 4) notes. However, as Sophia Reed (2018, p. 38-39) warns, “being a mom became my whole identity...Do not get so consumed in being a parent that a parent becomes your only identity.” Nevertheless, single mothers experience a sense of self-love and completion, as Amy Nickell (2018, p. 91) writes, “I also started to fall back in love with myself”.

Another indication of the discussed negotiation is the search for social justification through religion, as it is a well-established social institution. Religiosity remains a significant aspect of the self-identity of single mothers. Among the corpus of books analyzed, eleven of them discuss faith and spirituality as a crucial foundation in the authors’ lives, outnumbering the four devotionals. Only one author, Sarah Kowalski (2017), practices Buddhism, while the remaining authors identify as Christians.

***Semantic strategies.*** This identity negotiation leads to elaborating strategies, at least narrative, to elevate its ramifications and negotiate possible compromise. The semantic strategies are therefore key elements for writers / single mothers to sustain a fragile balance between challenging negative stereotypes and adjusting their unconventional families to fit into overall society. Here are the descriptions of seven sematic strategies.

1. The readers encounter conflict resolution strategies such as reframing or renaming, which are widespread in self-help literature ( Renegar & Cole, 2019). The most obvious example of reframing is negotiation of self-image during pregnancy without a partner. The identity negotiation that pregnant single mothers experience can be a difficult and confusing time. Not only are they grappling with changes in their bodies and the prospect of becoming a single parent, but they are also faced with societal expectations about how they should look and act during pregnancy. This can lead to feelings of shame and voicelessness, as they struggle to reconcile their physical limitations with the pressure to maintain a certain image, or as Sarah Kowalski (2017) puts it, “remain secretive, shamed by their body’s inability” (p. XV).

However, women are able to turn this tension into a positive experience. They see pregnancy as an opportunity to embrace their bodies and enjoy the physical experience of carrying a child. By reframing their experience in this way, they may find empowerment in the changes that are happening to their bodies. For some, this may even be a transformative experience, helping them to feel more comfortable in their own skin and more confident in their ability to handle the challenges of motherhood. At some point, single motherhood, especially at different stages of insemination and pregnancy, is regarded as a source of personal growth, unexpected benefits and even pleasure: “I think how I am happy to be doing this alone” ( Soiseth, 2008, p. 186), or “I wasn’t failure… I was, instead, an empowered woman choosing to have a baby from a highly conscious place” ( Kowalski, 2017, p. 84) or “...motherhood ... has given me a rudder” ( Kowalski, 2017 p. 254), or “Being single during this post-birth time was, for me, definitely a blessing in disguise” ( Nickell, 2018, p. 125).

Louise Sloan (2007) uses reframing to shift the focus onto the physical signs of pregnancy, which are often stigmatized by society, “I loved being pregnant... I loved being enormous, walking around in my yoga pants with my big belly hanging out. I loved that my body image was no longer an issue but an empowering experience” (p. 187). By embracing her body and refusing to be ashamed of her appearance, she was able to find empowerment in her pregnancy that went beyond the simple act of carrying a child. This is a powerful reminder that even in the face of societal pressure and expectations, women have the ability to redefine their experiences and find strength in their own bodies.

2. The renaming strategy, as a semantic approach, can be well illustrated by the use of positive descriptive adjectives. The extensive list of adjectives that writers use to describe themselves and their target audience, and therefore their implied readers, is rather long and indicates how renaming occurs. This procedure shows how single mothers attempt to replace negative stereotypes with socially positive traits by adding as many positive adjectives as possible. This focus on extensiveness explains the wide range of adjectives that are rarely repeated. However, there is consistency in the repetitive semantic fields across the books covered by the adjectives ‘normal’, ‘honest’, and ‘responsible’. The other non-repeated adjectives across the advice books are ‘attractive,’ ‘awesome,’ ‘brave,’ ‘consistent,’ ‘cool,’ ‘courageous,’ ‘creative,’ ‘flexible,’ ‘focused,’ ‘generous,’ ‘giving,’ ‘independent,’ ‘intellectual,’ ‘kind,’ ‘limitless,’ ‘loyal,’ ‘lovely,’ ‘patient,’ ‘proactive,’ ‘reasonable,’ ‘special,’ and ‘strong.’ Additionally, the assessment of single mothers’ features is realized through value-laden nouns such as survivor, fixer, miracle, and achiever, and expressions like “at the pinnacle of success” ( Sloan, 2007, p. 100), “the visionary leader” ( Farr, 2017, p. 131), and “the busiest, hardest-working people” ( Floch, 2014, p. 159).

In their attempt to strengthen achievements in the eyes of society through renaming, the authors expose themselves to neoliberal thinking. They apply a broad vocabulary related to entrepreneurial and business-like thinking: business genius, “successful CEO for your family and yourself” ( Whitehurst, 2010, p. 46), “the chief executive officer (CEO)” ( Farr, 2017, p. 47, 124-125), and “the most entrepreneurial group of people” ( Golds, 2014, p. 22), “a risk junkie” ( Golds, 2014 p. 70). The authors insist that single mothers possess and know how to implement personal agency and empowerment: “[Y]ou are in control of your own life” ( Isenhart, 2000, p. 72), or “You’ve got total responsibility, to be sure, but you’ve also got total control and freedom” ( Sloan, 2007, p. 31), or “Now, I’m not motivated by money, but I am motivated by being the best I can be” ( Golds, 2014, p. 14).

In addition to using descriptive adjectives, authors also engage in a process of reversing social vocabulary by replacing negatively nuanced words with their positive counterparts. This process is also aimed at challenging negative societal perceptions of single motherhood and presenting it in a positive light. For instance, Janis Adams (2011) suggests that single motherhood is often associated with a “curse” or a negative fate, but by recategorizing it as an opportunity for a “great future for both mom and kids,” she challenges this negative image and highlights the potential benefits of single motherhood.

3. The author seeks to normalize or routinize single motherhood by presenting it as comparable to other forms of motherhood and suggesting that it can be mundane. This is achieved by downplaying the difficulties and asserting that the experience of single motherhood has been less challenging than anticipated: “[S]ingle motherhood is just as beautiful to experience and just as potentially boring to talk about as any other kind of motherhood ... it’s been much easier than I expected it to be” ( Sloan, 2007, p. 235).

The author also uses the strategy of normalisation by emphasizing the common experience of pregnancy and childbirth, and by suggesting that being a mother is something that all women share. The statement, “I feel like I’m finally swimming in the same stream as every other woman” ( Soiseth, 2008, p. 144), is an expression of this strategy, as it highlights the sense of commonality and shared experience.

4. Narratively, the authors directly and indirectly address the social pressures and constraints imposed upon them. Direct confrontation involves rejecting false expectations created in their minds. For instance, Gina Panettieri ( Panettieri & Hall, 2008) negates the rooted images of single motherhood, stating “Single parenting is a lot better than people give it credit for!” (p. 88). Janis Adams (2011) echoes the sentiment in a more direct manner, “You are not a victim” (p. 73). Amy Nickell (2018) rebels against defaulted assumptions about single mother’s dependency on financial support from the father of the child by refusing to claim payment, “out of principle” ( Nickell, 2018, p. 98). Direct confrontation can also manifest as a conflict between failed expectations and acceptance of the positivity of unconventional family arrangements, as expressed by Alexandra Soiseth (2008): “I’m confused about how I feel, where that understanding fits in with my wanting to have a more traditional arrangement. It strikes me that maybe I don’t want that so much after all” (p. 214). Recent texts affirm the presence of single parents in society matter-of-factly, such as Ava Eagle hertz’s (2019) statement, “I wish we didn’t have single-parent homes, but unfortunately, this is inevitable” (p. 142). Ava Eagle Brown bluntly suggests stopping “questioning yourself” (2019, p. 11)

Indirect confrontations in single mothers’ narratives are a way to challenge commonly held beliefs about single motherhood without being too assertive. This can be achieved through the use of different figures of speech. For example, Sophia Reed (2018) uses a comparison and rhetoric question to challenge the way society views single mothers. In her book, she asks, "So how many of us are out here living like peasants, forgetting that we are princesses?" (p. 26). This highlights the fact that society often views single mothers as second-class citizens, when in fact they are just as valuable and deserving of respect as anyone else. By funnelling the conversation in this way, Reed challenges readers to rethink their assumptions about single motherhood.

Another indirect strategy to address stereotypes is to expose false expectations of society transmitted through pop culture. In many cases, pop culture perpetuates negative stereotypes of single mothers, portraying them either as unable to provide for their children or as superheroes who overcome any challenges on their ways. As a response, some authors use their narratives to expose these false expectations. For example, Gina Panettieri ( Panettieri & Hall, 2008) writes, “Americans, both adults and kids, loved the depiction of successful, inventive single moms creating a happy alternative family” (p. 85).

5. The authors employ social distinctions as a semantic strategy to reposition themselves in society, demonstrating their social worth and, in some cases, distinguishing themselves from other groups of single mothers. The narratives highlight the multifaceted nature of single motherhood, reaffirming its usefulness for society: “the ultimate authority in the household... the bearer of information, the provider, and the educator” ( Adams, 2011, p. 185-186); planner of the future ( Adams, 2011, p. 251); and keeper of the family heritage ( Smith, 2014, p. 87). Often, it involves the ability to accomplish multiple tasks simultaneously, which can be seen as a personal achievement: “I earned a Master’s degree; started a private counselling practice; took part in an overseas short-term mission trip; developed a writing and speaking ministry; and took part in the creation of a faith-based non-profit agency that serves single-parent families in the community” ( Floch, 2014, p. 216). This illustrates the positive impact single mothers can have on society by fitting into the patterns of a reliable and independent citizen. Logically, single mothers renegotiate their own status and the phenomena itself as a path to new opportunities with the “power of choice” ( Floch, 2014, p. 219).

Acknowledging the diversity within a community is a means of distinguishing oneself from other groups of single mothers. As Teresa Whitehurst (2010) points out, “we are not one monolithic entity. Single mothers differ from one another in attitudes, values, occupations, income, education, and priorities” (p. 20). The recognition of diversity within single motherhood not only allows a subgroup to distance themselves from others, but it also highlights the existence of a partially socially and partially self-imposed hierarchy. This distinction can be observed through the most well-known example of the term ‘single mother by choice’ coined by Jane Mattes, which aims to combat negative stereotypes about single motherhood ( Hertz, 2006; Mattes, 1997; Morrissette, 2008; Sloan, 2007). This creates a stratification within the community of single mothers, which can result in a sense of exclusion for those who do not identify with the shared label.

6. To navigate their unique experiences, single mothers develop their own language and conceptual framework, which they use to describe their “New World” ( Panettieri & Hall, 2008, p. 224). This language is a synthesis of common terms of kinship and their own recognizable phraseology, allowing them to express their experiences in a way that is both relatable and distinct. Through this process, they carve out a space for themselves that is separate from dominant societal narratives.

The authors recreate their unique language to describe their own support networks, which both overlap with and distance from existing societal institutions. They seek out other single mothers and form connections based on shared experiences, providing each other with emotional support and practical assistance. By doing so, they reject the traditional image of the isolated, helpless single mother and create a new narrative of strength and resilience.

The authors employ semantic strategies to redefine and elevate their status in society by coining new self-labelling words and expressions. For example, terms like “Chief Wellness Officer” and “Rock Stars” ( Smith, 2014, p. 84, 84), singlemumdom ( Golds, 2014, p. 181), mompreneur ( Brockes, 2018, p. 252) are used to positively self-identify and highlight their strengths. Additionally, some authors use other types of discourse practices, such as manifestos, to assert their agency and politically-artistically empower themselves. One such example is “The Kickass Single Mom Manifesto” by Emma Johnson, which outlines principles and values for single mothers to follow ( Johnson, 2017, p. 8–10). These language innovations serve to support a distinctive single mother identity, separate from the negative societal stereotypes surrounding single motherhood. By creating a unique language and discourse, single mothers are able to define themselves on their own terms and challenge the dominant narrative.

7. The balance between newly coined phraseology and recognizable terminology to designate kinship, extended family members, and members of single mothers’ community is essential to stay on the borderline between conformity and deviance from conventional family norms. This balance is crucial in the hope of being accepted by the community and family: “The Choice Mother must ensure that solo parenting does not become an isolating experience” ( Morrissette, 2008, p. 398). “A single mother cannot solely raise her children” ( Adams, 2011, p. 188), emphasizing the need for a support network beyond the individual. This strategy provides a more elaborate means of reinforcing the notion that single mothers are integrated into society while also differentiating themselves through their efforts to establish a support network that can compensate for the absence of a conventional two-parent family. They develop a distinct single mothers’ vernacular to describe their family replacement and network which is a combination of recognisable words, such as ‘family, ‘friend’, ‘partner’ and their semantically re-categorized peers, such as ‘mess’, ‘peeps’, ‘net’, ‘web’, ‘tribe’, ‘unit, ‘band’.

I have classified the terminology used to refer to the various members of a single mother’s support system into three main semantic subsets based on the level of kinship and emotional connections in their relationships. The semantic fields share similar features and reflect the strategies that authors use to describe their social surroundings. They combine the usage of both hypernyms and hyponyms. Notions that are difficult to semantically subdivide further may be extended by descriptive adjectives ^1^. Table 1 provides an overview of the three semantic subsets of single mothers' support system identified in this study, which were described in the previous section. The interplay of these strategies allows writers to imagine, describe, and expand their support system as broadly as their linguistic imagination permits.

**Table 1.** Three semantic subsets of single mothers’ support system.

(I) This semantic subset includes concepts for people who offer encouragement, empathy, and who are directly involved in the day-to- day care of the children and have a deep emotional connection with the single mother and her children through kinship or marriage:	
*Hypernyms:*	*Hyponyms:*
[collected / extended / home-built / immediate / wider] family (12); parents (12); [distant / trusted adult male] relatives (7); [extended / family / golden / social] circle (4); [loving / non-kin / other parent’s / trusted] family member (3); in-laws (2); matriarch (1).	uncle (12); aunts (10); mother (9); sister (9); cousin (7); brother (6); grandfather (6); father (5); grandparents (4); grandmother (3); [baby-loving] sister-in-law (3); stepfather (3); brother-in-law (2); children’s father (2); nephew (2); siblings (2); great-aunt (1); niece (1).
(II) This semantic subset encompasses individuals who offer practical assistance, such as childcare, transportation, and household chores, and who play a role in the single mother’s life but are not connected by kinship:	
*Hypernyms:*	*Hyponyms:*
[dream / supportive] team (7); [male / significant] role model (5); acquaintance (2), [non-related] caregiver (2); [adult / another / older / other / significant] males (2); significant other (2); sounding board (2); adult (1), [caring / good / responsible] man (1); church member (1); clergy (1); [caring / decent] human being (1); inner sanctum (1); male figure (1).	[a few good / best / close / close family / closest / college / entrepreneurial / Facebook / family / gay best / gay male / good / great / male / married / old high school / single / trusted / well-meaning / wise] friend(s) (25); other single mothers (16); neighbours (14); [responsible / tremendous / trustworthy] babysitter (11); [birthing / career / divorce / hockey / labour / leadership / life / sport / T-ball / transformational] coach (11); colleagues (7); other mothers (6); co-worker (5); nanny (5); other parents (5); child-minder (4); fellow churchgoers / worshippers (4); girlfriend (4); minister (4); other single parents (4); pastor (4); godparents (3); known donor / co-parent (3); sitter (3); au pair (2); other father (2); priest (2); church elder (1); guru (1); housemate (1); legal guardian (1); [future / current] lover (1); [spiritual] master (1); mates (1); pals (1); other fatherless teens (1); soulmate (1); spiritual guides (1); surrogate mother (1).
(III) This semantic subset includes people who offer different sorts of assistance or help with budgeting and time management, and more formal support systems that aid single mothers in areas such as housing, health care, well-being, and employment:	
*Hypernyms:*	*Hyponyms:*
[childcare / informal / online / professional / stronger] support network (13); [maternity / online / prenatal / secret / social / child’s peer] support group (11); [wider / intentional] community (8); [business / formal / older male] mentor (11); [financial / mental health / unbiased] professional (6); people down the street / [like-minded / positive / significant / successful] people (4); school staff (1); [family support] system (1).	[legal /family / professional / pastoral / school] counsellor (12); doctor (11); [day-care / male / school / spiritual / yoga pregnancy] teachers (10); therapist (10); [debt / financial] advisor (5); [accountability / birthing / business / ex- / father’s new / female / labour] partner (5); doula (4); lawyer (4); [boy Scout / club / male / youth] leaders (4); accountant (3); clients (3); [baby / school] nurse (3); paediatrician (3); [health / nursery / social / youth] workers (3); attorney (2); boss (2); customers (2); encourager (2); [mental health] facilitator (2); house cleaner (2); [recreation] instructors (2); [support / family] meditator (2); [certified financial / baby] planner (2); [healthcare / other care] provider (2); psychiatrist (2); solicitor (2); trainer (2); volunteers (2); advocate (1); bookkeeper (1); clinician (1); consultant (1); expert (1); fertility specialist (1); general practitioner (1); gynaecologist (1); handyman (1); [mother’s] helper (1); landlord (1); librarian (1); [good] listeners (1); married couples (1); matchmaker (1); midwife (1); physician (1); psychotherapist (1); preacher (1); spinster (1); supervisor (1); [local] teenager (1); tutor (1).

Note: I have included an exhaustive list of adjectives for each term in square brackets, along with the number of books that mention words in round brackets.

On an individual level the authors as well as their readers use these strategies to communicate more effectively with their larger society and create a clear and concise understanding of their social support system. By using hypernyms and hyponyms, they can convey both the general category and specific subcategory of each person in their support system. Descriptive adjectives can also provide more nuanced definition of the nature and quality of these relationships, helping to convey the level of emotional and practical support that each person provides. Additionally, authors invent expressions to describe their support networks using their own vernacular phraseology, such as ‘family-and-friend set’ (1), ‘too-bad-he’s-gay buddy’ (1), and the popular expression ‘it takes a village’ (6), to make the terms more intimate and familiar to themselves. By carefully choosing their words and using these sematic strategies, single mothers can express the diversity and complexity of their support system and emphasize the importance of these relationships in their lives.

The social level of these strategies is that they enable single mothers to navigate and create a sense of community within a society that often marginalizes or stigmatizes their family structure. By developing a distinct vocabulary to describe their support system, they are able to communicate their experiences and needs more effectively, both within their own community and to those outside of it. This can lead to a greater sense of validation and understanding, as well as facilitate the building of social support networks. Additionally, these linguistic strategies can also serve as a means of empowerment and resistance, as they allow single mothers to assert their agency and individuality in a society that often places a premium on traditional nuclear family structures.

## Conclusions

The growing number of advice books written by and for single mothers reflects a demand for positive and personalized narratives about their experiences, which have been highly stereotyped in the media. Although self-help books have been criticized for reinforcing repressive societal values about women, they also offer the potential for transformation of self-identity and social relationships. By allowing individuals to share their personal and intimate life experiences through an objectifying literary genre, these books serve the purpose of self-reflection and provide authors with a cultural instrument to step out of their insider position and become a narrating, judging outsider. As the single motherhood community continues to grow and evolve, self-help books can offer a powerful tool for resistance, self-empowerment, and the construction of a more personalized image of oneself.

The corpus of 30 self-help books presenting different demographics and paths to single motherhood was investigated using content analysis tools to discern single mothers’ semantic strategies applied to reinforce or challenge stereotypes, define subjectivity, and shape identity within the community. Semantic strategies, which encompass linguistic and rhetorical perspectives, discursive and narrative strategies, serve as an instrument to culturally construct, negotiate, and reframe single motherhood within at least the narratives of self-help literature. The analysis of these self-help books provides insight into the dynamics of self-perception that gained prominence in the second half of 2010s.

The ways these books characterize the experience of single mothers, even though in a constant attempt to dispel existing stereotypes, perpetuate a cultural narrative that extends the cultural privilege of biological parents, as well as white, heterosexual, Christian, wealthy women. The voices of divorced and by choice mothers who socially distinguish with their achievements on their previous stages of life sound lounder than those who do not have enough social capitals to defend themselves against prejudice.

Overall, the overrepresentation of white, Christian, heterosexual authors in the examined self-help books sheds light on both writers and their targeted audience, which primarily comprises white, privileged women. This homogeneity in authorship and readership suggests a potential lack of diverse perspectives and experiences, limiting the inclusivity of the self-help genre. Moreover, the prevalence of divorced single mothers among the writers reinforces the prominence of narratives cantered on biological privilege, potentially perpetuating narrow notions of family structures and societal roles. As a result, the semantic moves observed in the essay are primarily driven by privileged women, often from white backgrounds. This homogeneity in the perspectives presented can inadvertently reinforce existing power structures and societal norms, as seen in instances where CEO-based redefinition strategies or superwoman stereotypes are perpetuated.

In advice books single mothers centre narrative around rejection of the notion that their ability as parents is defined by their marital status. They prioritize being good parents over conforming to societal expectations of marriage and traditional family structures. They assert that a home without a father is not necessarily incomplete and emphasize the importance of instilling values in their children regardless of the family structure.

The authors convey conflicting messages that depict unrealistic expectations and opposing characteristics. These contradictions create a ground for a single mothers’ identity negotiation, where they struggle to define themselves as mothers and individuals while navigating societal expectations of what it means to be a mother and a single parent. Conflicting messages can also perpetuate negative stereotypes and stigmas about single mothers. The identity negotiation can manifest narratively through a multitude of questions that they are expected to answer in various ways, triggering a rediscovery of positive self or a reflective search for renewed and more comfortable self-identity. In several instances, the examples presented show that single mothers are reshaping or redefining their experiences in a manner that aligns with prevailing norms rather than outright rejecting them. The emergence of a distinct genre dedicated to these narratives hints at the presence of a potent norming function still influencing single motherhood – establishing a prescribed and “correct” way to be a single mother. The analysed self-help books work in tandem to shape the perception of single motherhood, portraying it within a particular framework. While some may employ subtle resistance to challenge existing norms, others may inadvertently perpetuate and solidify these norms. By examining the interplay between these narratives, we can better grasp the complex dynamic at play – one that may simultaneously resist and reinforce societal expectations. The exploration of this nuanced perspective would offer a deeper understanding of how these books, as a collective force, influence the societal discourse surrounding single motherhood. Their ability to reshape experiences within the context of established norms opens the door to both possibilities of empowerment and potential entrenchment of limiting narratives.

The content analysis allowed me to discern seven principal semantic strategies that the authors of self-help books apply in their texts: reframing, renaming, normalization or routinization, direct or indirect confrontations, distinctions, self-labelling, and vernacular.

Reframing involves changing the way a person or community is perceived by emphasizing different aspects or redefining the experience in a more positive light. In the context of single motherhood, reframing is used to shift the focus from negative aspects to more positive ones, empowering women and helping them to find strength and confidence in their own bodies, despite societal pressure and expectations.

The renaming strategy involves using positive adjectives and value-laden nouns to redefine the experience of being a single mother in a more positive light. This is achieved by replacing negative stereotypes with socially positive traits, emphasizing personal agency and empowerment, and challenging negative societal perceptions of single motherhood. The authors of advice books for single mothers use extensive vocabulary related to entrepreneurial and business-like thinking, and they aim to present single motherhood as an opportunity for success and personal growth. They also engage in a process of reversing negative societal vocabulary by using positive counterparts.

Normalization or routinization means presenting single motherhood as comparable to other forms of motherhood and downplaying its difficulties. This approach emphasizes the common experience of pregnancy and childbirth, suggesting that being a mother is something all women share, and that single motherhood can be seen as a mundane part of that shared experience.

Direct confrontation involves rejecting false expectations and affirming the positivity of unconventional family arrangements, while indirect confrontation challenges commonly held beliefs about single motherhood through the use of figures of speech and exposing false expectations perpetuated through pop culture. The authors aim to challenge readers to rethink their assumptions about single motherhood.

Social distinctions are used to position single mothers in society and demonstrate their social worth. They highlight the multifaceted nature of single motherhood and its usefulness for society. Single mothers renegotiate their status and the phenomena of single motherhood as a path to new opportunities. Acknowledging the diversity within the community is a means of distinguishing oneself from other groups of single mothers, and this can result in a sense of exclusion for those who do not identify with the shared label. The term “single mother by choice” is an example of creating a stratification within the community of single mothers.

Single mothers develop their own language and conceptual framework to describe their experiences and create a space for themselves that is separate from dominant societal narratives. They seek out other single mothers to form connections and reject the traditional image of the isolated, helpless single mother. They use coining new self-labelling words and expressions to positively self-identify and highlight their strengths, as well as other types of discourse practices. By creating a unique language and discourse, single mothers can define themselves on their own terms and challenge the dominant narrative.

Single mothers use a combination of recognizable vocabulary and their own vernacular to describe their support networks and navigate their unique experiences. The balance between conforming to conventional family norms and differentiating themselves is important in creating a sense of community. They use hypernyms, hyponyms, and descriptive adjectives to communicate effectively about the emotional and practical assistance they receive from their support system, and also create expressions that are intimate and familiar to themselves. By doing so, they can assert their agency and individuality, challenge negative stereotypes surrounding single motherhood, and build social support networks.

By employing positive language to describe single motherhood, authors attempt to reshape societal attitudes and promote a more positive perception of this experience. This strategy can be effective in challenging negative stereotypes and empowering single mothers. It also contributes to the larger societal conversation around single motherhood, which can help to shape policies and programs that better support single mothers and their families.

This research underscores the importance of listening to and amplifying the voices of single mothers, who often face marginalization and erasure in dominant cultural narratives. Moreover, it highlights the potential of linguistics and discourse analysis to offer nuanced and contextualized understandings of individual and collective experiences, as well as to inform and shape public policies and social interventions.

Moving forward, it is essential to continue exploring the diverse semantic strategies employed by single mothers in different contexts, and to examine how these strategies intersect with other dimensions of identity, such as race, ethnicity, sexuality, and class. This can help to generate a more comprehensive and inclusive understanding of the experiences of single mothers and contribute to the development of more effective and equitable support systems. By centering the experiences and perspectives of single mothers, we can work towards building a society that values and empowers all individuals and families, regardless of their structures or circumstances.

## Data Availability

*All data underlying the results are available as part of the article and no additional source data are required*.
