# Supplementary material for: Dynamics of single mothers’ semantic strategies in Anglophone non-fiction
Source: Open Res Eur. 2023 Aug 8;3:79. Originally published 2023 May 16. [Version 2] doi: 10.12688/openreseurope.15729.2 (PMC10446034; doi:10.12688/openreseurope.15729.2)
Supplement: Peer review history

# Peer review: Reviewer response for version 2

**DOI:** 10.21956/openreseurope.17790.r34340

**Published:** 2023-08-14

**Authors:** Sucharita Sarkar

**Affiliations:** 1 D.T.S.S College of Commerce, Mumbai, Maharashtra, India

No more comments.

If any, are all the source data and materials underlying the results available?

Yes

Is the work original in terms of material and argument?

Yes

Is the work clearly and cogently presented?

Yes

Is the argument persuasive and supported by evidence?

Yes

Does the research article contribute to the cultural, historical, social understanding of the field?

Yes

Does it sufficiently engage with relevant methodologies and secondary literature on the topic?

Yes

Reviewer Expertise:

Motherhood studies

I confirm that I have read this submission and believe that I have an appropriate level of expertise to confirm that it is of an acceptable scientific standard.

# Peer review: Reviewer response for version 1

**DOI:** 10.21956/openreseurope.16993.r32558

**Published:** 2023-06-22

**Authors:** Sucharita Sarkar

**Affiliations:** 1 D.T.S.S College of Commerce, Mumbai, Maharashtra, India

The article studies 30 self-help books (advice literature) written by single mothers during the period 1990 to 2020. It deploys critical discourse analysis to map seven semantic strategies (reframing, renaming, normalization, direct or indirect confrontations, distinctions, self-labelling, and vernacular) in these texts which map how these single mothers contest, navigate and reframe existing pejorative stereotypes about single motherhood into more affirmative and empowering experiences and identities.

 The brief but relevant review of literature provided by the author exposes that although scholars have focused on the self-help genre before, not much work has been done on this specific subset of self-help books by and about single mothers. Hence, this article is a fresh and welcome attempt to bridge the gap.

 The critical discourse analysis methodology is objective and data-driven, and I find it appropriate for analysis the large corpus of 30 books that were selected by the author, especially within the limitations of an article (as opposed to a larger research project on this same data set).

 The argument is lucidly and cogently presented and adequately supported by quotes from the selected texts. The inclusion of the table identifying and classifying support systems used by single mothers is particularly useful and effective. Space permitting, I would have urged for a larger number of tables that demonstrated the application of the research methodology to the texts selected, especially for semantic strategies such as renaming and self-labelling.

 I have two suggestions. Firstly, a disclaimer that these 30 books, in spite of the plurality of their authors' identities, are mostly written by 'single mothers by choice,' and does not include the struggles and stigma encountered by many other categories of single mothers by chance (for instance, teenage single mothers). Secondly, the article does not attempt to address the underlying contradictions and depoliticization of neoliberal resilience narratives such as self-help books: the fact that the shift of focus on the resilient mother and her chosen network of family and friends often omits necessary demands for structural changes in state and societal support that feminists and feminist mothers have been working towards (see for instance, *Mothering in the Age of Neoliberalism* edited by Melinda Vandenbeld Giles, or T *he Aftermath of Feminism: Gender, Culture and Social Change* by Angela McRobbie).

 In spite of these absences (which are understandable owing to space limitations), overall, I believe the article adds new nuances to the understanding of motherhood and mothering in contemporary times, especially focusing on the experiences of single mothers of choice.

If any, are all the source data and materials underlying the results available?

Yes

Is the work original in terms of material and argument?

Yes

Is the work clearly and cogently presented?

Yes

Is the argument persuasive and supported by evidence?

Yes

Does the research article contribute to the cultural, historical, social understanding of the field?

Yes

Does it sufficiently engage with relevant methodologies and secondary literature on the topic?

Yes

Reviewer Expertise:

Motherhood studies

I confirm that I have read this submission and believe that I have an appropriate level of expertise to confirm that it is of an acceptable scientific standard.

# Peer review: Reviewer response for version 1

**DOI:** 10.21956/openreseurope.16993.r32563

**Published:** 2023-06-16

**Authors:** Jai Mackenzie

**Affiliations:** 1 Department of Humanities, Newman University, Birmingham, England, UK

This article presents an overview of the content and strategies used in a selection of self-help books focused on single motherhood. It makes a valuable contribution to understanding of the ways in which single motherhood, as well as norms of parenting and family life more generally, are negotiated in this genre. The analysis and discussion offer a broad overview of the content, messages and strategies of the texts. I am in agreement with all of the comments and revisions suggested by the first reviewer, Valerie Renegar, and would particularly emphasise the suggestion that the author bring more nuance into their discussion of the texts’ complex negotiations of social norms, and also that they account for the privilege of the books’ authors and audiences. I would also add the following suggestions for revision, which focus mainly on the methodology and article structure:

1. Through the course of the article, the diverse groups which can fall under the category ‘single mother’ are drawn out. It would be useful to outline this more clearly from the outset, in the ‘introduction’, so that the reader has a clear sense of what is meant by a ‘single mother’, and what groups the article is focusing on, from the start.
2. In the ‘data collection’ section, it would be useful to know a little more about the books - a table, with the title of each other, would be useful. This would be preferable to listing the books as citations – see additional point on citations below (point 5). The detail currently in the ‘results’ could also be brought into this section, as you do present a more thorough overview of the books here (and this overview does not really constitute the ‘results’ of your analysis).
3. In the ‘data analysis’ section, it is noted that a critical discourse analytical framework is used. However, the nature of CDA, or the ways in which it is applied to this analysis, are not really explained. I would suggest that the analysis does not really constitute CDA, and that it is also not particularly linguistic in approach. It may be more appropriate to frame the analytical approach in terms of a) content analysis of the books’ coverage, and b) identification of the authors’ discursive and rhetorical strategies If CDA is particularly significant to the methodology, this should be explained, with reference to relevant sources, and should be evident throughout the analysis and discussion.
4. The ‘discussion’ section (which might be more aptly named ‘analysis and discussion’), is divided into two sub-sections: ‘identity crisis’ and ‘semantic strategies’. The latter section is very logically constructed, outlining the 7 strategies you have identified, with examples. The function of the first section is less clear: I think this is perhaps intended as an overview of how the authors negotiate social norms in relation to their own lives – if so, this may be largely an issue of naming (see point in Renegar’s review on the term ‘identity crisis’). In any case, it would be useful to signpost the purpose of each section more clearly.
5. Data excerpts and citations: I would advise making a clear distinction between data and secondary literature throughout the article (also see point 2, above). In the ‘discussion’ section, strategies for referring to data differ; sometimes examples are quoted, whilst at other times a page reference is given. Here I would advise listing examples from the data clearly as quotations in the form of excerpts, and reserving the citation format exclusively for secondary literature.
6. The similarities across the self-help texts come across very strongly, but there is little mention of any differences. Consideration of difference, alongside the points raised by the first reviewer, would bring nuance and depth to your analysis. This would be particularly relevant in relation to the diversity of ‘single mothers’ across the authors and readership.

If any, are all the source data and materials underlying the results available?

Yes

Is the work original in terms of material and argument?

Yes

Is the work clearly and cogently presented?

Partly

Is the argument persuasive and supported by evidence?

Yes

Does the research article contribute to the cultural, historical, social understanding of the field?

Yes

Does it sufficiently engage with relevant methodologies and secondary literature on the topic?

Partly

Reviewer Expertise:

Motherhood and family studies, language and gender, language and new media, discourse analysis (critical and poststructuralist)

I confirm that I have read this submission and believe that I have an appropriate level of expertise to confirm that it is of an acceptable scientific standard, however I have significant reservations, as outlined above.

# Peer review: Reviewer response for version 1

**DOI:** 10.21956/openreseurope.16993.r31695

**Published:** 2023-06-08

**Authors:** Valerie Renegar

**Affiliations:** 1 Department of Communication Studies, Southwestern University, Georgetown, Texas, USA

This essay is a critical textual analysis of a large sample of self-help books aimed at single mothers. The author isolates the rhetorical devices that are employed in these texts in order to discern how single mothers are rhetorically constructed by this genre. They are: reframing through conflict resolution, ne-naming experiences and relationships, normalizing alternative family models and roles, the identification of social pressure, negotiating social worth, linguistic innovation, and re-definition and utilization of terminology. Textual examples of each of, as well as critical discussion, are used to illustrate each of the strategies. Ultimately, the author concludes that the books " *perpetuate a cultural narrative that extends the cultural privilege of biological parents, as well as white, heterosexual, Christian, wealthy women*" even as they are attempting to resist the social expectations about families.

 I find this to be an engaging and well written manuscript, and I appreciate adding this dimension to our collective understanding of the changing face of motherhood. I have four main suggestions for revision.

1. In the Discussion, you open with the idea of identity crisis. I encourage you to rethink this particular term. “Crisis” conveys a sense of stress, urgency, and trauma, and while some of the semantic terms and themes isolated later are about developing a sense of identity, these women don’t seem to be in crisis as much as they are in a state of negotiation and redefinition. Thinking about this section as “Identity negotiation” or “Defining identity” removes some of the negativity associated with crisis and makes the findings of the study more relevant to that goal. You return to this idea of an identity crisis in the Conclusion, but again there are more productive and appropriate ways to describe this process of redefinition.
2. Whiteness is mentioned a few times throughout the essay, but a more thoroughgoing discussion of the racial elements of single-motherhood, and the audience to whom these books are targeted is relevant and would productively influence the findings. Similar to Renegar and Cole’s work with stepmothers that you cite in the paper, it seems that these books are aimed at a tiny, privileged audience. Most of the single mothers in the US are non-white, and the vast majority have not earned a college degree ( Pew Research, 2018), which means that they are also more likely to be poor. Self-help books, however, tend to be consumed by white, privileged women. So, the semantic moves described in the essay can only be described as the moves made by privileged women who are usually white. This is especially clear in the examples of CEO-based redefinition strategies or superwoman stereotypes. There is very little discussion of privilege or class in the manuscript, but this, too, would help better frame who writes these books and who consumes them. The claim in the conclusion that " *The ways these books characterize the experience of single mothers, even though in a constant attempt to dispel existing stereotypes, perpetuate a cultural narrative that extends the cultural privilege of biological parents, as well as white, heterosexual, Christian, wealthy women.*" rings true based on my experience, but there is very little discussion of these elements in the analysis itself. A more robust development of these elements (biological privilege, whiteness, sexual orientation, religion, and class) would enhance the critical value of the essay.
3. In the 'Identity Crisis' section, you initially claim that self-help authors resist social norms, but I would encourage you to think more critically about this claim. In the following paragraphs, you discuss the contradictions implicit in doing motherhood differently while still adhering to the expectations of motherhood.  This section would be enhanced with a brief discussion about the outsized influence of the regime of intensive mothering and the disciplining function it has for mothers.
4. In the 'Identity Crisis' section, and again in the conclusion, you claim single mothers “ *resist existing social norms and construct their own definitions of family and parenthood*.” However, your analysis indicates that this is only sometimes true. In some of the examples you have provided, the women are reframing or redefining their experience so that it conforms to the existing norms rather than actively rejecting those norms. The fact that these books form their own genre suggests that there is a powerful norming function still at work here - a way to do single motherhood “right”. Considering making this claim more nuanced would allow for you to discuss the ways that these books work together to define single motherhood in a particular way, and how they might be used to resist norms but may also serve to entrench them further.
5. One much more minor suggestion, the last paragraph of the Introduction terms single motherhood as " *a transitional stage in life*" that occurs post divorce. This phrase seems to beg the question of what these women are transitioning to. Do most single mothers remarry or later cohabitate? What makes this stage transitional instead of stable?

If any, are all the source data and materials underlying the results available?

No source data required

Is the work original in terms of material and argument?

Yes

Is the work clearly and cogently presented?

Yes

Is the argument persuasive and supported by evidence?

Yes

Does the research article contribute to the cultural, historical, social understanding of the field?

Yes

Does it sufficiently engage with relevant methodologies and secondary literature on the topic?

Yes

Reviewer Expertise:

Feminist theory, motherhood studies, rhetoric and rhetorical theory

I confirm that I have read this submission and believe that I have an appropriate level of expertise to confirm that it is of an acceptable scientific standard, however I have significant reservations, as outlined above.
